# Comparative Pharmacokinetics of Berberine, Palmatine and Jatrorrhizine in Rat Plasma after Oral Administration of *Rhizoma coptidis* and Zuojinwan Using Liquid Chromatography-Tandem Mass Spectrometry

**Published:** 2012

**Authors:** Rui Yan, Yin Wang, Youping Liu, Xin Di

**Affiliations:** *School of Pharmacy, Shenyang Pharmaceutical University, Shenyang, China. *

**Keywords:** *Rhizoma coptidis*, Zuojinwan preparation, Liquid chromatography tandem mass spectrometry, Pharmacokinetics

## Abstract

A selective and sensitive liquid chromatography-tandem mass spectrometry (LC-MS/ MS) method was developed for the determination of berberine, palmatine and jatrorrhizine in rat plasma. Target compounds, together with the internal standard (metronidazole), were extracted from rat plasma samples by protein precipitation with acetonitrile-methanol (1:2, v/v). Chromatography was carried out using a C_18 _column (150 × 4.6mm, 5μm) under isocratic elution with water (containing 0.3% formic acid)-acetonitrile (30:70, v/v). The mass spectrometric detection was performed by selected reaction monitoring (SRM) mode via electrospray ionization (ESI) source operating in positive ionization mode. The method was linear over the concentration range of 0.2-100 ng/mL for all components. The intra- and inter-day precision values were less than 14.7% and the deviations were within ± 9.0%. The validated method was applied to the comparative pharmacokinetic studies of berberine, palmatine and jatrorrhizine after oral administration of *Rhizoma coptidis *and Zuojinwan. The results indicated that the pharmacokinetics of berberine, palmatine and jatrorrhizine were significantly different between different groups.

## Introduction

Recipe is the soul of traditional Chinese medicine (TCM). In the clinical practice of TCM, multiple herbs often combine to produce new pharmacological activities. The composite formulae will produce a synergistic effect or antagonistic action in the body. 

**Figure 1:**
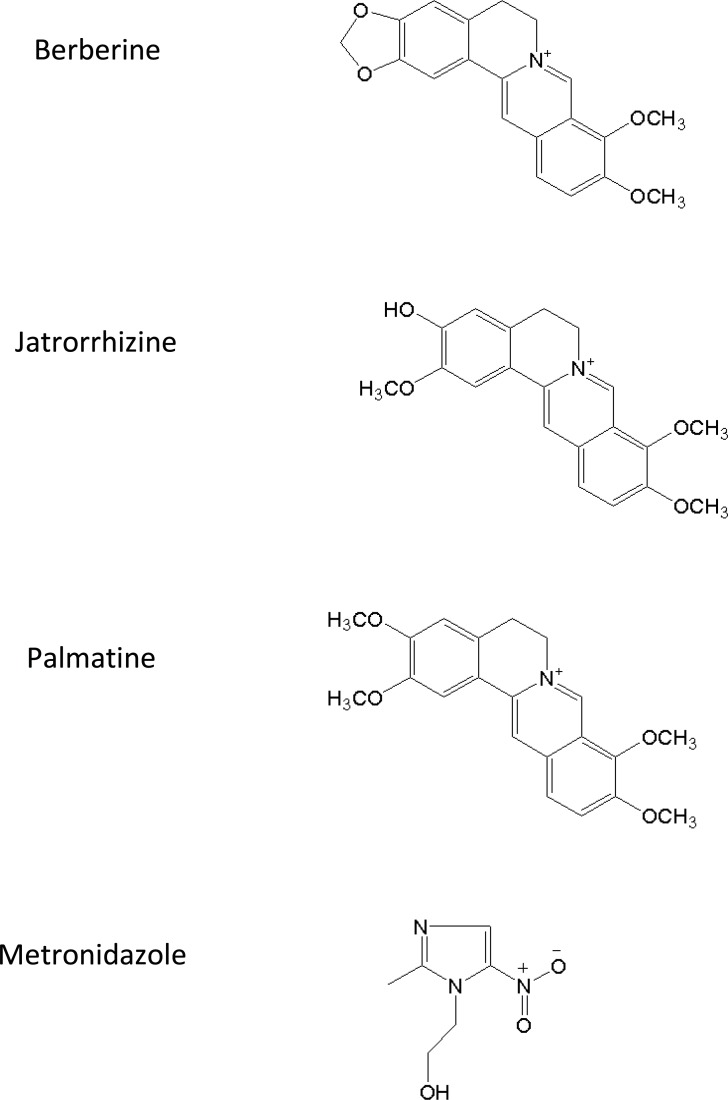
Chemical structure of berberine, jatrorrhizine, palmatine and metronidazole (I.S.).

*Rhizome coptidis *(1-3) and *Evodia rutaecarpa *(4, 5) have been widely used for centuries in TCM due to broad therapeutic effects. As a typical couple, various ratios of combinations of *Rhizoma coptidis *and *Evodia rutaecarpa *can produce diverse pharmacological effects. Zoujinwan, which consists of *Rhizoma coptidis*-*Evodia rutaecarpa *powder (6:1, g/g), has been used to treat gastro-intestinal disorders in the clinical practice of TCM with a long history. Berberine, palmatine and jatrorrhizine are regarded as the most important pharmacologically active constituents. Their chemical structures were shown in Figure 1. 

Previous studies have developed methods to investigate the pharmacokinetics of berberine, palmatine and jatrorrhizine after administration of monomers or herbs (6-11). However, there was no comparative study on the prescription to analyze the mechanism of the combination. In this paper, a sensitive and selective method of liquid chromatography-electrospray ionization-mass spectrometry (LC-ESI-MS) is presented for the simultaneous determination of berberine, palmatine and jatrorrhizine in rat plasma. This assay was then applied to an intercomparsion pharmacokinetic study of the three constituents after oral administration of *Rhizoma coptidis *and Zuojinwan in rats. 

## Experimental

*Chemicals and reagents*

Berberine (purity 98.5%), palmatine (purity 98.0%) and jateorrhizine (purity 99.0%) were purchased from the National Institute for the Control of Pharmaceutical and Biological Products (Beijing, China). Methanol, formic acid, acetonitrile were of chromatographic grade from the Yuwang Chemical Factory (Shandong, China). Deionized water was purified by use of an Alpha-Q water-purification system (Millipore, Bedford, MA, USA) for the preparation of samples and buffer solution. All other reagents were of analytical grade. *Rhizoma coptidis *and *Evodia rutaecarpa *were purchased from the Sifang Pharmacy (Shenyang, China).

*Instrumentation conditions*

The HPLC system consisted of a LC-10ADvp Pump (Shimadzu, Kyoto, Japan) and a SIL-HTA Autosampler (Shimadzu, Kyoto, Japan). Chromatographic separation was carried out on a Diamonsil C_18_ column (150 × 4.6 mm, 5 μm, Dikma) with a EasyGuard C18 Security guard column (8 × 4.0 mm I.D., Dikma) kept at 20°C. The mobile phase consists of water (containing 0.3% formic acid) : acetonitrile (30 : 70, v/v), at a flow rate of 0.45 mL/min.

Mass spectrometric detection was performed on a Thermo Finnigan TSQ Quantum triple quadrupole mass spectrometer (San Jose, CA, USA) equipped with an ESI source in the positive ionization mode. The MS operating conditions were optimized as follows: the spray voltage: 4200 v; the heated capillary temperature: 320°C; the sheath gas (nitrogen): 30 Arb; the auxiliary gas (nitrogen): 5 Arb; the collision gas (argon) pressure: 1.2 mTorr. Data acquisition was performed by Xcalibur 2.0 software. Peak integration and calibration were performed using LCquan software. Quantification was obtained by using SRM mode of the transitions at *m/z *336→320 for berberine, at *m/z *352→336 for palmatine, at *m/z *338→322 for jatrorrhizine and at *m/z *172→128 for metronidazole (IS) respectively, with a scan time of 0.3 s per transition.

*Preparation of the standard and quality control (QC) samples*

A mixed stock solution containing 100 μg/mL of berberine, palmatine and jateorrhizine was prepared in methanol. A series of working standard solutions were prepared by successive dilution of the mixed stock solution with methanol. A 200 ng/mL I.S. working solution was similarly prepared by diluting a stock standard solution of metronidazole with methanol. Calibration standards were prepared by spiking 100 μL of the appropriate standard working solutions into 50 μL blank plasma to yield calibration concentrations of 0.2, 0.4, 1.0, 4.0, 10.0, 40.0, 100.0 ng/mL for each component. QC samples were prepared at 0.4, 4, 80 ng/mL for each component.

*Sample preparation*

Rat plasma 50 μL was mixed with 50 μL internal standard solution (200 ng/mL), 150 μL methanol and 100 μL acetonitrile. After vortex-mixing 2 min, the mixture was centrifuged at 10 krpm for 5 min. The supernatant was separated out and blown to dryness with nitrogen at 40°C. Then the residue was reconstituted in 100 μL mobile phase and a 10 μL aliquot of the final testing samples was injected onto the LC-MS system for analysis.

*Method validation*

The method was validated according to the currently accepted USA Food and Drug Administration (FDA) bioanalytical method validation guidance.

Method linearity was evaluated by analyzing calibration standards in duplicate at each concentration level over three consecutive days. The accuracy and precision were assessed by analyzing QC samples in six replicates at three concentration levels on three validation days. The extraction recovery was evaluated at three concentration levels and for the I.S. at one concentration level by comparing the peak areas of the analytes obtained from six plasma samples with the analytes spiked before and after extraction. Matrix effect was evaluated by comparing the peak areas of the analytes obtained from six plasma samples with the analytes spiked after extraction, at three concentration levels, to those for the neat standard solutions at the same concentrations. The stability of the analytes in rat plasma at low and high concentration levels was evaluated under a variety of storage and process conditions.

*Pharmacokinetic application*

Five Male Sprague-Dawley rats (250 ± 20 g) were fasted for 12 h. The rats were split into two groups to complete the crossover design for pharmacokinetic experiment with a washout period of 7 days. Each rat was administered an oral dose of herb powders suspended in an aqueous solution containing 0.5% carboxymethyl cellulose sodium (1.08 g *Rhizome coptidis *powder/kg body weight). 150 μL blood samples were collected in heparinized Eppendorf tubes via the oculi chorioideae vein before dosing (0 min) and subsequently at 10, 20, 45, 90, 150, 180, 210, 300, 420, 480, 720 and 1440 min after administration. The heparinized blood was immediately centrifuged for 5 min at 12 Krpm, and the plasma obtained was stored at -20°C until analysis. The pharmacokinetic parameters were calculated using a non-compartmental analysis using DAS 2.0 software (Mathematical Pharmacology Professional Committee of China, Shanghai, China).

## Results and Discussion

*Method development*

**Figure 2:**
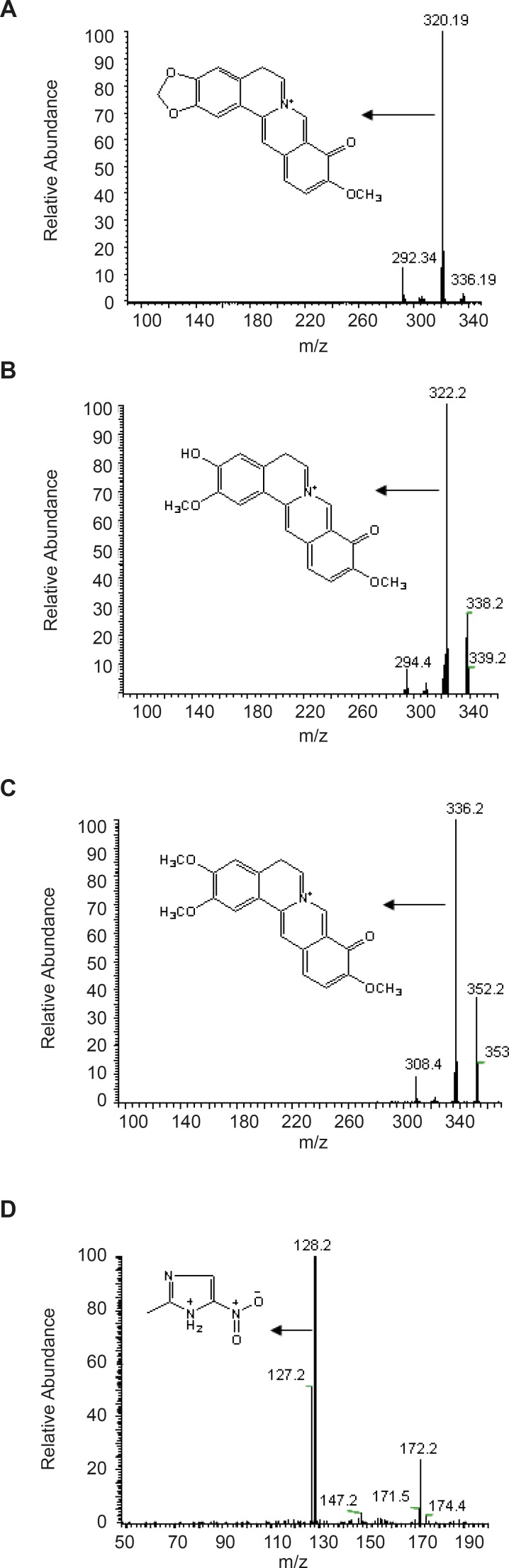
Product ion mass spectra of [M]^+ ^ions of (A) berberine (B) jatrorrhizine (C) palmatine (D) I.S.

To obtain maximum sensitivity of the SRM, some parameters such as spray voltage, source CID, sheath gas (nitrogen) pressure, auxiliary gas (nitrogen) pressure, collision gas (argon) pressure, and collision energy were optimized. The other MS parameters were adopted from the recommended values for the instrument. The typical full-scan ESI mass spectrum of analytes and I.S. is described in Figure 2.

The mobile phase played a critical role in achieving good chromatographic behavior and appropriate ionization. The selected mobile phase was composed of 30% water (containing 0.3% formic acid) and 70% acetonitrile and provided low background noise and proper retention time.

*Method validation*

**Figure 3:**
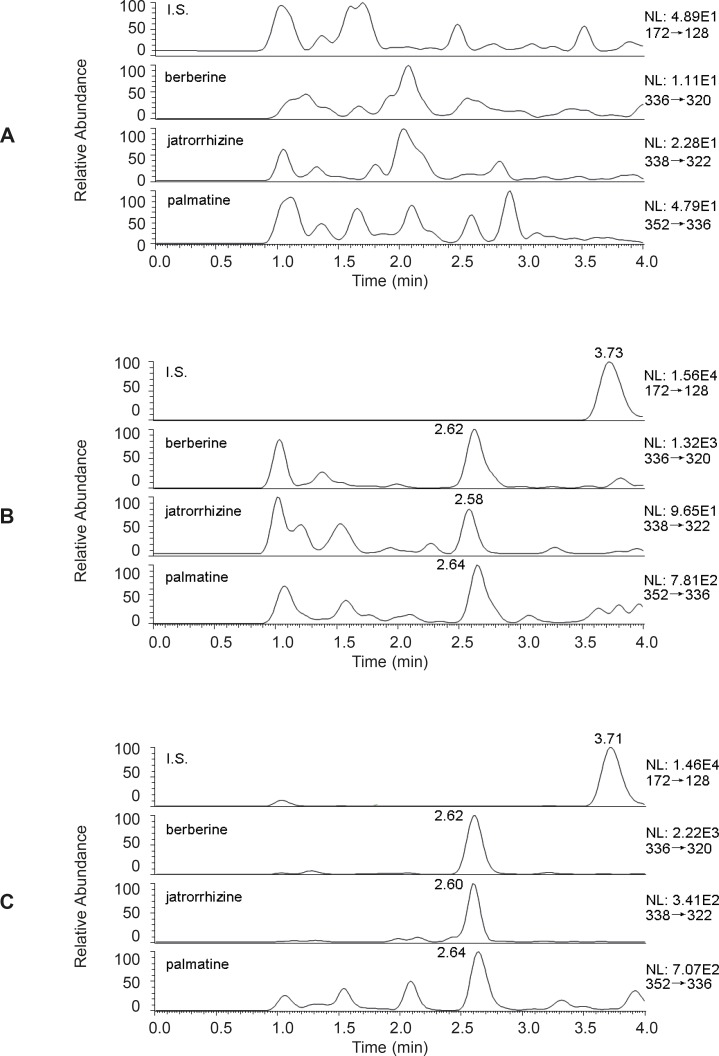
Representative SRM chromatograms for analytes in (A) a blank plasma sample (B) a blank plasma spiked with standard at the LLOQ, I.S. at 200 ng/mL (C) a plasma sample after administration of Zuojinwan

The typical chromatograms of a blank, a spiked plasma sample with berberine, palmatine and jatrorrhizine (0.2 ng/mL) and I.S. (200 ng/mL) and plasma obtained 180 min after oral administration of Zuojinwan were presented in Figure 3. All samples were found to be of no interference at the retention times of the analytes or the I.S.

Typical equations of the calibration curve using weighted (1/x^2^) least squares linear regression were as following: y = 0.23x+0.027, *r*^2^ = 0.9930 (berberine), y = 0.028x-0.0026, *r*^2^ = 0.9833 (jatrorrhizine), y = 0.086x+0.024, *r*^2^ = 0.9927 (palmatine). All calibration curves showed excellent linearity over the range 0.2-100 ng/mL in rat plasma. Table 1 contained the precision and accuracy data corresponding to LLOQ, the intra- and inter-day precision and accuracy data and the mean extraction recoveries for berberine, palmatine and jatrorrhizine. The mean extraction recovery of the I.S. was 75.7 ± 10.2%. Table 2 summarized the stability data of QC samples. The results showed that all the samples were stable during these tests and there were no stability related problems during the routine analysis of samples for the pharmacokinetic study. The data displayed in Table 3 indicated that the co-eluting matrix components had little or no effect on the ionization of the analytes and IS. 

**Table 1:** Intra- and inter-day precision and accuracy for determination of berberine, jatrorrhizine and palmatine in rat plasma

**compound**	**Added**	**Found**	**(%)RSD**		**Relative error**	**% Recovery**	
	**ng/mL**	**ng/mL**	**Intra-day**	**Inter-day**	**%**	**mean**	**SD**
**Berberine**	0.2	0.218	6.8	/	9.0		
	0.4	0.42	6.2	10.7	5.0	95.2	7.8
	4	3.99	8.6	13.5	-0.25	87.9	4.8
	80	84.85	8.6	10.4	6.06	92.6	4.2
**Jatrorrhizine**	0.2	0.202	14.7	/	1.1		
	0.4	0.40	11.0	6.2	0.3		12.1
	4	3.95	11.6	1.6	-1.25	79.2	11.7
	80	84.89	6.6	14.5	6.11	87.3	5.8
**Palmatine**	0.2	0.215	6.1	/	7.6		
	0.4	0.40	11.1	3.4	1.2	84.0	5.4
	4	4.13	8.2	11.2	3.25	75.1	6.4
	80	85.11	8.9	14.3	6.4	90.9	4.9

**Table 2:** Stability data of berberine, jatrorrhizine and palmatine in rat plasma under various storage conditions (n = 3).

**Storage condition**	**Spiked concentration (ng/mL)**	**Calculated concentration **(**mean ± SD**) (ng/mL)				**RE (%)**		
		**berberine**	**jatrorrhizine**		**palmatine**	**berberine**	**jatrorrhizine**	**palmatine**	
Room temperature (4 h)	0.4	0.44 ± 0.01		0.41 ± 0.04	0.42 ± 0.03	10.0	2.5	5.0	
	80	78.89 ± 8.01	82.31 ± 9.38		78.04 ± 10.88	-1.4	2.9	-2.4	
Freeze and thaw	0.4	0.39 ± 0.05	0.41 ± 0.03		0.42 ± 0.03	-2.5	2.5	5.0	
	80	69.20 ± 3.22	77.63 ± 6.94		69.67 ± 6.87	-13.5	-3.0	-12.9	
Autosampler (24 h)	0.4	0.40 ± 0.04	0.40 ± 0.05		0.41 ± 0.03	0.0	0.0	2.5	
	80	71.17 ± 4.47	72.21 ± 2.00		67.08 ± 1.83	-11.0	-9.7	-16.2	

**Tabble 3:** Matrix effect data for berberine, jatrorrhizine and palmatine at 3 levels of QC samples and IS 200 ng/mL in six different sources of rat plasma

**compound**	**(Nominal plasma concentration **(**ng/mL**	**(Matrix Effect **(**%**)(**mean ± SD**	**%RSD**
**Berberine**	0.4	4.6 ± 85.6	5.4
	4	11.2 ± 89.9	12.5
	80	2.1 ± 98.2	2.2
**Jatrorrhizine**	0.4	10.6 ± 77.5	13.7
	4	10.7 ± 89.6	11.9
	80	4.4 ± 82.9	7.0
**Palmatine**	0.4	12.0 ± 85.4	14.0
	4	13.9 ± 102.8	13.5
	80	7.1 ± 94.1	7.6
**.I.S**	200	13.3 ± 92.9	14.3

*Results of pharmacokinetic study*

**Figure 4:**
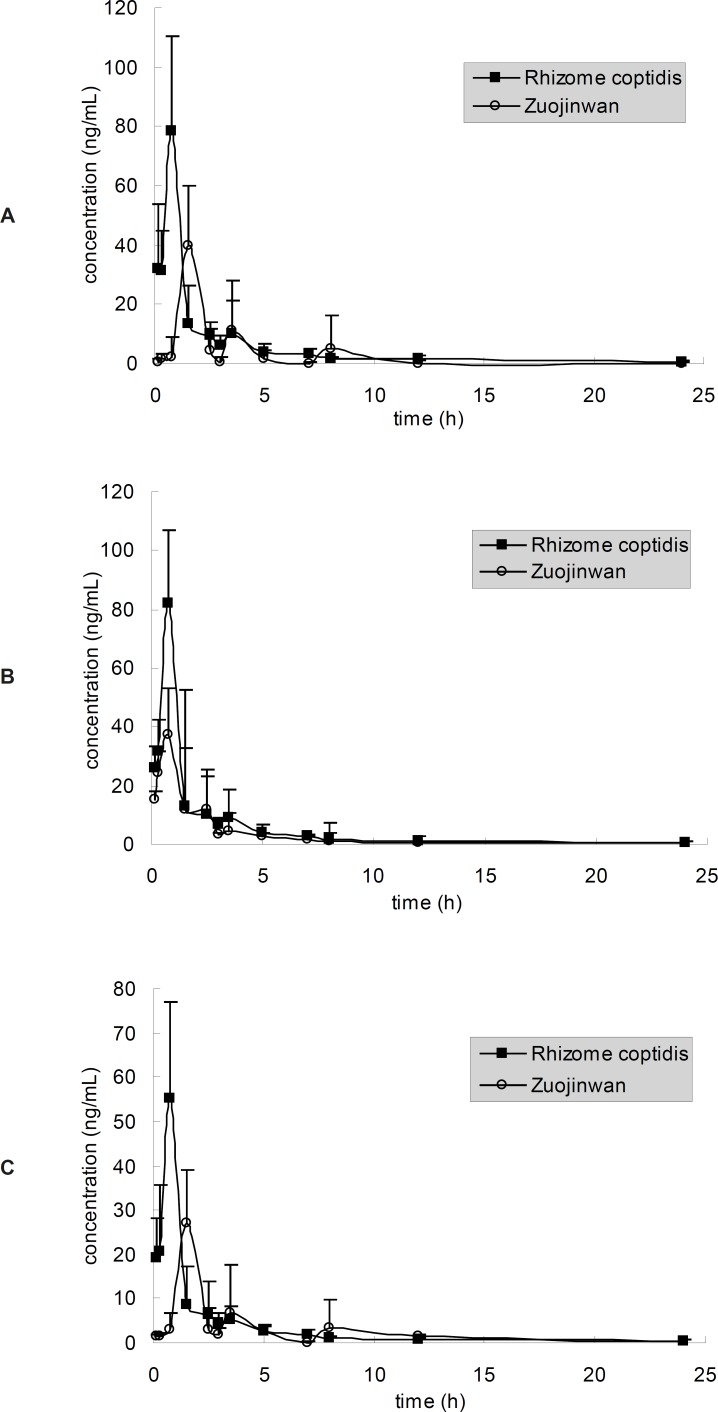
Mean plasma concentration-time curves of (A) berberine (B) jatrorrhizine and (C) palmatine after oral administration of Rhizome coptidis and Zuojinwan

The method described above was applied to the pharmacokinetic study in which plasma concentrations of berberine, palmatine and jatrorrhizine were determined for 24 h after oral administration of *Rhizome coptidis *and Zuojinwan (1.08 g *Rhizome coptidis *powder/Kg body weight). The mean plasma concentration-time profiles (n = 5) were represented in Figure 4. Berberine, palmatine and jateorrhizine exhibited similar plasma concentration–time profiles under the same administration methods, which probably due to the similarity of their molecular structures. The similarity was observed in many studies on TCM (12-14). Probably the similarity of their molecular structures contributed to their similar disposition process *in-vivo*. Pharmacokinetic parameters are listed in Table 4. The parameters were very different with the results in (7), probably because the pharmacokinetic experiments are so susceptible that the pharmacokinetic data would often differ when the experimental conditions, such as animals, herbs and sampling time, change a little.

**Table 4:** Mean pharmacokinetic parameters of berberine, jatrorrhizine and palmatine in rat plasma (n = 5).

**Compound**	**p. o.**	**AUC**_0-t_ **(μg/L·h)**	**AUC**_0-∞_ **(μg/L·h)**	**MRT**_0-t_ **(h)**	**MRT**_0-∞_ **(h)**	**t**_1/2z_ (h)	**T**_max_ (h)	**C**_max_ **(μg/L)**
Berberine	*Rhizoma coptidis*	126.8 ± 28.1	133.5 ± 31.4	3.8 ± 0.9	5.4 ± 1.8	7.2 ± 1.8	0.75 ± 0.11	78.42 ± 12.19
	Zuojinwan	69.7 ± 52.9	70.8 ± 56.5	3.7 ± 0.9	4.1 ± 1.7	4.4 ± 4.1	1.50 ± 0.89	40.06 ± 12.15
Jatrorrhizine	*Rhizoma coptidis*	123.1 ± 31.1	128.9 ± 37.4	3.5 ± 0.8	4.9 ± 3.8	7.1 ± 6.4	0.75 ± 0.11	82.09 ± 17.44
	Zuojinwan	107.9 ± 50.8	113.8 ± 48.1	4.3 ± 0.9	5.9 ± 3.0	8.0 ± 3.7	1.50 ± 0.89	39.63 ± 13.35
Palmatine	*Rhizoma coptidis*	78.8 ± 18.1	81.4 ± 18.2	3.2 ± 0.8	4.1 ± 1.0	6.2 ± 1.4	0.75 ± 0.11	55.35 ± 8.90
	Zuojinwan	60.9 ± 33.8	62.2 ± 34.8	5.2 ± 1.1	5.7 ± 1.4	4.2 ± 2.3	1.50 ± 0.22	27.03 ± 6.57

Three peaks at 90, 210 and 480 min were observed in the plasma concentration-time curves after administration of Zuojinwan (Figure 4). The similar result had happened in Deng›s study (7). They discussed that distribution, re-absorption and enterohepatic circulation might contribute to multiple blood concentration peaks of berberine, palmatine and jateorrhizine after oral administration of Zuojinwan. Furthermore, we surmised that *Evodia rutaecarpa *could affect the dissolution rate from herb powders, which would limit the absorption rate of alkaloids. It would take more time for the constituents to dissolve out of deep layer than surface layer of herb powders, as a result, causing the appearance of the second and the third blood concentration peaks in Figure 4. In addition, Zuo *et al. *(8) reported that jatrorrhizine is one of the metabolites of berberine, we can infer that the second and third peaks may be caused by the newly produced jatrorrhizine which was metabolized from berberine. Similarly, berberine and palmatine are also possibly metabolites of some other constituents in herbs. The second and third peaks may be caused by the newly produced alkaloids which were metabolized from other alkaloids. *Evodia rutaecarpa *probably exacerbated the metabolism of the alkaloids in *Rhizoma coptidis*.

The pharmacokinetic curves of the same constituent were different under different administration methods. There was a single peak of the alkaloids in the pharmacokinetic curves after oral administration of *Rhizoma coptidis, *which was different with that of Zuojinwan. It must be the components in *Evodia rutaecarpa *that caused the multiple peaks. Earlier publications have also reported multiple blood concentration peaks after administration of herbs or preparations (15, 16). According to Table 4, the t_max_ after administration of Zuojinwan is longer than that of *Rhizoma coptidis*, the C_max_ after administration of Zuojinwan is lower than that of *Rhizoma coptidis*, the AUC after administration of Zuojinwan is a little lower than that of *Rhizoma coptidis*. *Evodia rutaecarpa *could moderate the potency and prolong the action time of *Rhizoma coptidis*. “Essentials of Matea Medica”, an ancient TCM book, explained that the warm nature of *Evodia rutaecarpa *could attenuate the bitter cold nature of *Rhizoma coptidis *so that protect the stomach from the repulsion between illness and medicines.

In conclusion, the method is sensitive and selective for the simultaneous determination of berberine, palmatine and jatrorrhizine in rat plasma. This is the first report on the comparative pharmacokinetic study of berberine, palmatine and jatrorrhizine in Zuojinwan. The interactions between *Rhizoma coptidis *and *Evodia rutaecarpa *caused obvious alteration of the pharmacokinetic profiles of the alkaloids. *Evodia rutaecarpa *weakened the effects of *Rhizoma coptidis*. The results could provide important information for explaining the mechanism of Zuojinwan.

## References

[1] Tanaka T, Metori K, Mineo S, Hirotani M, Furuya T, Matsumoto H, Satoh T, Kobayashi S (1991). Studies on collagenase inhibitors. IV. Inhibitors of bacterial collagenase in Coptidis rhizome. Yakugaku Zasshi.

[2] Bova S, Padrini R, Goldman WF, Berman DM, Cargnelli G (1992). On the mechanism of vasodilating action of berberine: possible role of inositol lipid signaling system. J. Pharmacol. Exp. Ther.

[3] Chiou WF, Yen MH, Chen CF (1991). Mechanism of vasodilatory effect of berberine in rat mesenteric artery. Eur. J. Pharmacol.

[4] Lu YX, Lin XY, Lu ZY (2002). Chemical constituents of Evodia rutaecarpa and its clinical application. Pharm. J. Chin. PLA.

[5] Zhou LD, Li JC (2005). Reviews of pharmacology studies on Evodia rutaecarpa. Chin. Arch. Tra. Chin. Med.

[6] Lu T, Liang Y, Song J, Xie L, Wang GJ, Liu XD (2006). Simultaneous determination of berberine and palmatine in rat plasma by HPLC–ESI–MS after oral administration of traditional Chinese medicinal preparation Huang-Lian-Jie-Du decoction and the pharmacokinetic application of the method. J. Pharmaceut. Biomed.

[7] Deng YT, Liao QF, Li SH, Bi KS, Pan BY, Xie ZY (2008). Simultaneous determination of berberine, palmatine and jatrorrhizine by liquid chromatography-tandem mass spectrometry in rat plasma and its application in a pharmacokinetic study after oral administration of coptis-evodia herb couple. J. Chromatogr. B.

[8] Zuo F, Nakamura N, Akao T, Hattori M (2006). Pharmacokinetics of berberine and its main metabolites in conventional and Pseudo germ-free rats determined by liquid chromatography/Ion trap mass spectrometry. Drug Metab. Dispos.

[9] Yu S, Pang XY, Deng YX, Liu L, Liang Y, Liu XD, Xie L, Wang GJ, Wang XT (2007). A sensitive and specific liquid chromatography mass spectrometry method for simultaneous determination of berberine, palmatine, coptisine, epiberberine and jatrorrhizine from Coptidis Rhizoma in rat plasma. Int. J. Mass Spectrom.

[10] Huang J, Wang G, Jin Y, Shen T, Weng W (2007). Determination of palmatine in canine plasma by liquid chromatography– tandem mass spectrometry with solid-phase extraction. J. Chromatogr. B.

[11] Feng J, Xu W, Tao X, Wei H, Cai F, Jiang B, Chen WS (2010). Simultaneous determination of baicalin, baicalein, wogonin, berberine, palmatine and jatrorrhizine in rat plasma by liquid chromatography-tandem mass spectrometry and application in pharmacokinetic studies after oral administration of traditional Chinese medicinal preparations containing scutellaria–coptis herb couple. J. Pharm. Biomed. Anal.

[12] Wang W, Li CY, Wen XD, Li P, Qi LW (2009). Simultaneous determination of 6-gingerol, 8-gingerol, 10-gingerol and 6-shogaol in rat plasma by liquid chromatography–mass spectrometry: Application to pharmacokinetics. J. Chromatogr. B.

[13] Yang Z, Zhu W, Gao S, Xu HY, Wu BJ, Kulkarni K, Singh R, Tang L, Hu M (2010). Simultaneous determination of genistein and its four phase II metabolites in blood by a sensitive and robust UPLC–MS/MS method: Application to an oral bioavailability study of genistein in mice. J. Pharm. Biomed. Anal.

[14] Wen J, Hong ZY, Wu YW, Wei H, Fan GR, Wu YT (2009). Simultaneous determination of rupatadine and its metabolite desloratadine in human plasma by a sensitive LC-MS/MS method: Application to the pharmacokinetic study in healthy Chinese volunteers. J. Pharm. Biomed. Anal.

[15] Tsai PL, Tsai TH (2004). Hepatobiliary excretion of berberine. Drug Metab. Dispos.

[16] Li YH, Duan JP, Guo TT, Xie W, Yan SN, Li B, Zhou YQ, Chen YX (2009). In-vivo pharmacokinetics comparisons of icariin, emodin and psoralen from Gan-kang granules and extracts of Herba Epimedii, Nepal dock root, Ficus hirta Yahl. J. Ethnopharmacol.

